# Superior Mesenteric Artery Syndrome: An Infrequent Complication of Scoliosis Surgery

**DOI:** 10.1155/2014/263431

**Published:** 2014-10-22

**Authors:** Metin Keskin, Turgut Akgül, Adem Bayraktar, Fatih Dikici, Emre Balık

**Affiliations:** ^1^General Surgery Department, Istanbul Faculty of Medicine, Istanbul University, Capa, Millet Caddesi, 34093 Istanbul, Turkey; ^2^Orthopedic Department, Istanbul Faculty of Medicine, Istanbul University, Capa, Millet Caddesi, 34093 Istanbul, Turkey; ^3^General Surgery Department, School of Medicine, Koç University, Rumelifeneri Yolu, Sarıyer, 34450 Istanbul, Turkey

## Abstract

Superior mesenteric artery syndrome is a rare condition that causes a proximal small intestinal obstruction due to contraction of the angle between the superior mesenteric artery and the aorta. Scoliosis surgery is one of the 15 reasons for superior mesenteric artery syndrome, which can present with acute or chronic manifestations. Although conservative treatment is usually possible, surgical treatment is required in certain cases that cannot be treated using conservative methods. In this paper, we describe a patient who developed superior mesenteric artery syndrome after scoliosis surgery and was treated with duodenojejunostomy due to failure and complications of conservative treatment.

## 1. Introduction

Superior mesenteric artery (SMA) syndrome, first defined by von Rokitansky and referred to as SMAS by Wilkie, is a rare condition resulting from increased pressure generated by contraction of the angle between the SMA and the aorta, near the third part of the duodenum [1, 2]. Approximately 15 causes have been described for SMAS; scoliosis repair surgery is one of them [3–5]. In particular, after correction of the vertebral axis in scoliosis surgery, the angle between the SMA and the aorta can become narrow, which can cause obstruction of the duodenum [6]. SMAS can present with acute manifestations, such as a proximal small intestinal obstruction, or chronic manifestations, such as weight loss, vomiting, decreased appetite, and postprandial abdominal pain manifestations [3–5]. Conservative treatment is possible with parenteral nutrition and nasogastric tube decompression [7]. However, in rare cases, the conservative methods fail, and surgical treatment is required [8–10]. This report discusses the treatment course and outcomes of a patient who developed SMAS after scoliosis surgery and was treated with duodenojejunostomy because of the failure of conservative treatment.

## 2. Case Report

The physical examination of a 17-year-old female patient who presented to our orthopedics polyclinic complaining of a protrusion on her back revealed kyphosis and a stooped right shoulder, a right thoracic curvature with a left-facing opening, a 6 cm high right thoracic hump on the front leaning test, and shoulder asymmetry. However, no deficits were identified in the patient's neurologic examination. Adolescent idiopathic thoracic scoliosis (Type 1B according to the Lenke classification) was diagnosed via height measurements taken in the standing position [11].

**Figure 1:**
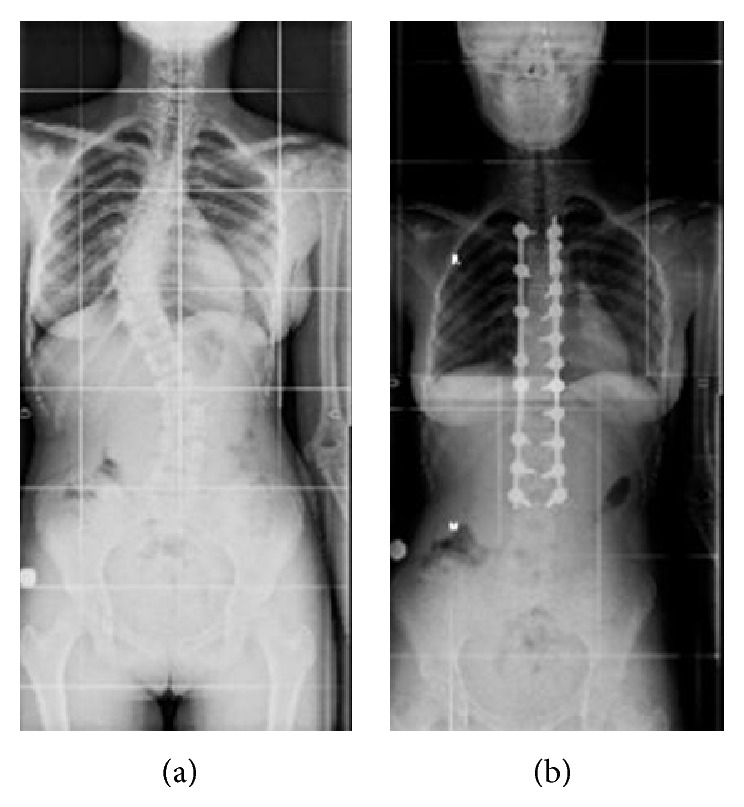
(a) Using preoperative radiography, thoracic scoliosis with right curvature was observed. (b) Postoperative radiography showing complete resolution of the spinal curvature after surgery.

**Figure 2:**
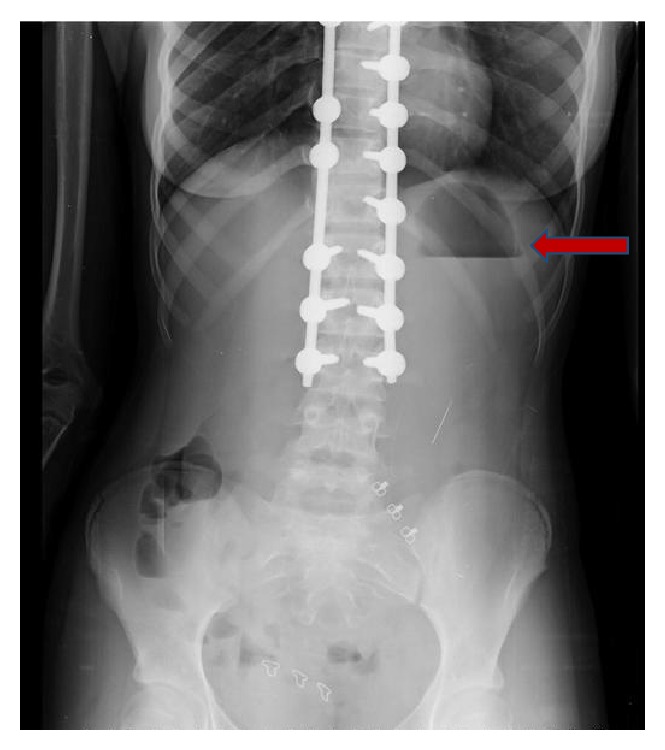
Air-fluid level in the stomach.

**Figure 3:**
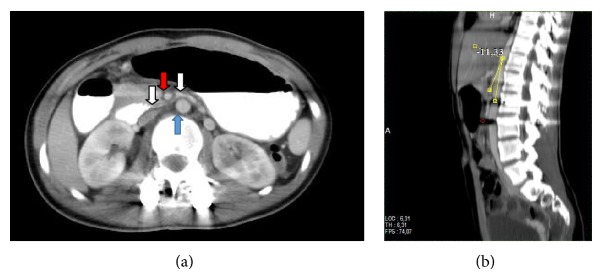
(a) Duodenum (white arrows) compressed between the aorta (blue arrow) and the superior mesenteric artery (red arrow). (b) The angle between aorta and SMA (11°).

**Figure 4:**
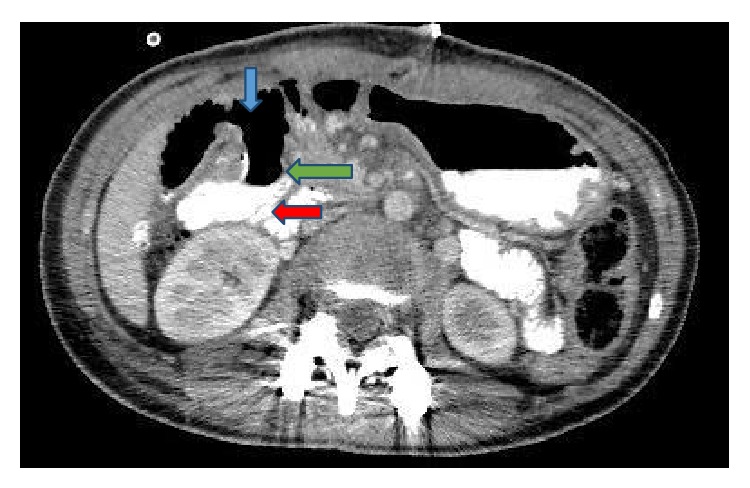
Oral contrast passing from the duodenum (red arrow) to the jejunum (blue arrow) and the patent duodenojejunal anastomosis (green arrow).

In the frontal plane, the measured Cobb angles were 50° in the thoracic region and 30° in the lumbar region [12]. The patient's bone development was classified as Risser Grade 3. She opted for surgical treatment, and her scoliosis was corrected with global derotation; distraction and compression were achieved with the use of titanium polyaxial pedicle screws between the T3–L3 vertebrae in the prone position. Additionally, 60 cc allografts were placed for fusion. No abnormalities were observed during intraoperative neurological monitoring. The patient was observed in the intensive care unit for the first 12 hours after the surgery, and complete resolution of the spinal curvature was noted on radiographs obtained 24 hours postoperatively (Figures 1(a) and 1(b)). She was then allowed to ambulate. The patient, who had no problems during the postoperative follow-up, was discharged from the hospital on the third day. On the fifth postoperative day, she presented to the orthopedic clinic with complaints of nausea, vomiting, and abdominal distention and was readmitted to the hospital. She was initially treated with nasogastric tube decompression but continued to have approximately 1500 cc of bile drainage daily, which did not decrease during treatment. General surgery consultation was therefore required, and an air-fluid level was identified in the stomach on direct abdominal radiography (Figure 2). She had experienced 4 kg of weight loss in the last week, and her weight was 39.5 kg when she was admitted to the surgical clinic. An obstruction that appeared compatible with outside pressure and that did not permit the endoscope to pass the 3rd part of the duodenum was identified during upper gastrointestinal endoscopy. Intravenous and oral contrast-enhanced abdominal tomography studies were therefore performed, which showed that the stomach and the first and second parts of the duodenum were significantly dilated. The aortomesenteric angle was narrowed, and the duodenum between the aorta and the SMA was nearly completely compressed. The angle between aorta and SMA measured 11 degrees and the patient was therefore diagnosed with SMAS (Figures 3(a) and 3(b)). She was treated conservatively with total parenteral nutrition and continued nasogastric decompression. However, there was no improvement in her symptoms after one week of conservative treatment. A second oral and intravenous contrast-enhanced CT scan showed persistent abnormalities. Therefore, the patient was treated surgically. Because the patient and her parents did not consent to laparoscopic surgery, after performing Kocher maneuver, antecolic, hand sewn, and side-to-side duodenojejunostomy was performed by open surgery. She had no complications during the immediate postoperative period and passed gas on the second postoperative day. Oral liquid nutrition was started on the third day after surgery. Oral contrast-enhanced abdominal tomography was performed on the fifth postoperative day due to abdominal pain; the anastomosis was patent, with intact transit of the intestinal contents (Figure 4). The patient, who was tolerating full nutrition on the seventh postoperative day, was discharged from the hospital without any further problems. She had no complaints during the initial 8 months of follow-up. Additionally, she gained 10.5 kg, to a total weight of 50 kg.

## 3. Discussion

SMAS is a rare condition resulting from increased pressure that is generated by contraction of the angle between the SMA and the aorta, near the third part of the duodenum. This condition was first defined by von Rokitansky [1] in 1861 and was referred to as SMAS by Wilkie [2] in 1927. Hence, SMAS is also known as Wilkie Syndrome. Its precise incidence is not known; however, barium swallow studies have suggested that the rate of SMAS is between 0.013 and 0.78% [7, 13]. After separating from the aorta, the SMA courses anteroinferiorly at an angle of approximately 45° (38–56°). The third part of the duodenum passes from right to left in the opening between the SMA and the aorta. If this angle contracts for any of the aforementioned reasons, the duodenum is squeezed between the SMA, the aorta, and the vertebral column. Consequently, complete or partial obstruction can occur in the third part of the duodenum [14, 15]. Approximately 15 causes of SMAS have been identified, including the following: a narrow SMA-aorta distance or a narrow aortomesenteric vascular angle; high fixation of the duodenojejunal flexure to the ligament of Treitz; a relatively low SMA origin; excessive weight loss (or other secondary causes of mesenteric and retroperitoneal lipid tissue loss, such as burns, anorexia, or cancer); severe injuries, such as head trauma; surgical complications; abnormal peritoneal attachments associated with duodenal malrotation; excessive lumbar lordosis; visceroptosis; and a loose abdominal wall [3–5]. Additionally, there are case reports in the literature concerning SMAS as a complication of scoliosis surgery [4, 6, 16–19]. Zhu and Qiu [4] associated this condition with a contraction of the angle between the SMA and the aorta, resulting from an extension of the SMA trunk that occurs after correction of the vertebral axis in scoliosis surgery. The degree of narrowing can result in acute or chronic clinical manifestations. The acute syndrome manifests as a proximal small intestinal obstruction, whereas the chronic form presents as weight loss, vomiting, decreased appetite, and postprandial abdominal pain [20]. The decreases of aortomesenteric fat tissues due to prolonged vomiting and weight loss can lead to vicious circle or increase of symptoms [4, 6]. In our case, the symptoms of SMAS appeared as a proximal small intestinal obstruction on the fifth day after vertebral surgery for scoliosis in a young woman. Here, we associated the development of SMAS with an insufficient amount of para-aortic lipid tissue, most likely related to the patient's cachexia, and with contraction of the angle between the aorta and SMA due to improvement of the vertebral axis after the spinal surgery. Zhu and Qiu [4] reported SMAS in 7/640 patients who underwent scoliosis operations. They reported that the symptoms appeared 5–7 days after surgery. This finding is consistent with our case, as the symptoms of nausea and vomiting developed on the fifth day after the surgery.

It is difficult to diagnose SMAS because there are no specific symptoms and because the condition is rare. Hence, certain diagnostic methods should be used. Dilation of the stomach can be observed by direct radiography, and obstruction of the duodenum, with proximal dilation, can be identified with a barium swallow series [8, 20, 21]. In addition, gastrointestinal endoscopy is necessary to eliminate the possibility of intraluminal pathologies [8, 13]. Doppler ultrasonography and angiography are also used to evaluate the aortomesenteric angle [9, 21, 22]. Laparoscopic diagnosis has also been reported [8, 14]. However, currently, the most efficient method for diagnosis is oral and intravenous contrast-enhanced abdominal tomography and angiography. Using this method, the duodenum and the vascular structures can be evaluated simultaneously [21, 23, 24]. In our patient, an air-fluid level was identified in a direct abdominal radiographic study. Pathologies in the bowel lumen were eliminated by upper gastrointestinal endoscopy, and outside pressure on the distal second part of the duodenum was observed. The diagnosis was made by double-contrast abdominal tomography.

Treatment of SMAS depends on whether the condition is acute or chronic, as well as on its etiology. Because the condition is rare, the diagnosis can be delayed. Case reports in the literature have described air infiltration into the duodenal wall and the portal vein, in addition to gastric and duodenal dilation, on CT scans that were obtained because of abdominal pain and vomiting [25, 26]. In SMAS, conservative treatment with internal and parenteral supportive care, including nasogastric tube decompression, can result in improvement [7, 20, 25, 26]. We treated our patient with nasogastric tube decompression and parenteral nutrition for 7 days; however, surgery was required because there was no response to this treatment. In order to avoid septic complications such as anastomotic leakage and wound infection which may be caused by malnutrition, it is necessary to start parenteral nutrition whether SMA is acute or chronic condition [6, 19]. Furthermore, it must be considered that “refeeding syndrome” which includes some electrolyte and metabolic imbalance following beginning of the enteral or parenteral nutrition after prolonged starvation can be seen in this patient [27].

The most preferred methods for the surgical treatment of SMAS are dividing the ligament of Treitz and extending the distance between the aorta and the duodenum (Strong's procedure) or duodenojejunostomy. Duodenojejunostomy is favored over gastrojejunostomy to protect pyloric function [8–10]. One case report has also described infrarenal transposition of the SMA as a new treatment method [23]. Minimally invasive methods (such as laparoscopic duodenojejunostomy) are also frequently used in SMAS [21, 28]. In our case, the patient and her relatives refused laparoscopy; we therefore performed a laparotomy with release of the ligament of Treitz and a single-layer, side-to-side duodenojejunostomy.

We found several case reports describing patients who developed superior mesenteric artery syndrome after scoliosis surgery in the PubMed database. All of the patients except one were treated conservatively [4, 6, 16–19]. In the current study, our patient underwent duodenojejunostomy due to the ineffectiveness of conservative treatment.

In conclusion, SMAS is a rare condition that is caused by the rotation of anatomic structures following scoliosis surgery and the consequent contraction of the aortomesenteric angle and extension of the SMA trunk. SMAS should be considered in patients who develop a proximal small intestinal obstruction after scoliosis surgery. The diagnosis can be made by contrast-enhanced abdominal tomography and upper gastrointestinal endoscopy. In most cases, symptoms are improved with conservative treatment. Otherwise, release of the ligament of Treitz (if possible with a laparoscopic approach) and duodenojejunostomy are ideal surgical treatment options for patients who do not respond to conservative treatment. Notably, delayed treatment of SMAS may result in death [18, 29].
